# When Fruit Turns Harmful: Late Diagnosis of Hereditary Fructose Intolerance in a Pediatric Patient—A Case Report and Literature Review

**DOI:** 10.1155/carm/9866803

**Published:** 2026-05-26

**Authors:** Michelle Higuera Carrillo, Sara Vallejo Tamayo, Melquisedec Vargas Sandoval

**Affiliations:** ^1^ Department of Pediatrics, Faculty of Medicine, National University of Colombia, Bogota, Colombia, unal.edu.co; ^2^ Department of Pediatrics, Hospital Universitario Infantil de San Jose, Fundacion Universitaria de Ciencias de la Salud (FUCS), Bogota, Colombia; ^3^ Department of Pediatrics, Universidad CES, Hospital Pablo Tobon Uribe, Medellin, Colombia, ces.edu.co; ^4^ Department of Gastroenterology, Gastrokids IPS, Pereira, Colombia

**Keywords:** *ALDOB* gene, Aldolase B deficiency, case report, fructose-free diet, hepatomegaly, hereditary fructose intolerance, hypoglycemia

## Abstract

**Introduction:**

Hereditary fructose intolerance is a rare but potentially severe and fatal disorder if it is not recognized promptly. It is caused by biallelic mutations in the *ALDOB* gene, which encodes the Aldolase B enzyme. Deficiency leads to intracellular accumulation of fructose‐1‐phosphate, causing secondary inhibition of gluconeogenesis and glucogenolysis and, consequently, hypoglycemia along with hepatic and renal dysfunction. The clinical presentation is variable and often nonspecific, making diagnosis challenging.

**Case report:**

A 12‐year‐old boy, born small for gestational age, with a history of hypotonia, laryngomalacia, and persistent hepatomegaly. During his first year of life, he developed an episode of acute hepatitis that required admission to a high‐dependency unit due to acute liver failure. Over the following years, he experienced recurrent episodes of vomiting, pallor, diaphoresis, and malaise following the ingestion of sweet foods. During follow‐up, he developed syncope secondary to severe hypoglycemia during a fructose tolerance test. Genetic testing revealed a homozygous pathogenic variant in the *ALDOB* gene, confirming the diagnosis of HFI. A strict diet excluding fructose, sucrose, and sorbitol was initiated, with close multidisciplinary follow‐up.

**Conclusion:**

HFI represents a diagnostic challenge in pediatrics due to its clinical heterogeneity and its ability to mimic multiple hepatic and metabolic diseases. This case highlights the importance of considering HFI in patients with unexplained gastrointestinal and hepatic symptoms, even beyond infancy. Increased awareness and early diagnosis are critical to prevent misdiagnosis, avoid metabolic decompensation, and ensure excellent long‐term outcomes.

## 1. Background

Hereditary fructose intolerance (HFI) is an autosomal recessive inborn error of carbohydrate metabolism caused by biallelic mutations in the *ALDOB* gene, located on chromosome 9q31.1. This gene encodes Aldolase B, a key hepatic enzyme in the fructolysis pathway responsible for catalyzing the reversible cleavage of fructose‐1‐phosphate (F1P) into glyceraldehyde and dihydroxyacetone phosphate [1].

Deficiency or dysfunction of Aldolase B leads to toxic intracellular accumulation of F1P in hepatocytes, renal tubules, and enterocytes. This accumulation exerts a potent inhibitory effect on both gluconeogenesis and glycogenolysis, causing severe metabolic disturbances such as profound hypoglycemia, lactic acidosis, hyperuricemia, and progressive hepatorenal dysfunction [2].

Clinically, symptoms emerge shortly after the introduction of fructose‐, sucrose‐, or sorbitol‐containing foods, typically between 4 and 6 months of age, when complementary feeding begins. Exposure to these sugars triggers the accumulation of the aforementioned metabolite, leading to postprandial vomiting, lethargy, sweating, hepatomegaly, and transient jaundice. If unrecognized or untreated, HFI may progress to chronic liver disease, renal impairment, and growth failure, and aversion to sweet foods, which may paradoxically delay diagnosis [3].

Despite advances in diagnostic methods, HFI remains unrecognized, particularly in patients with mild, atypical, or nonspecific symptoms. Misdiagnosis as infectious hepatitis, functional gastrointestinal disorders, or endocrine conditions is not uncommon, especially in early life. Although genetic testing of the *ALDOB* gene is currently considered the diagnostic gold standard, clinical suspicion and careful temporal correlation with dietary exposure remain essential, particularly in low‐resource settings where advanced testing may not always be available.

Therapeutic management relies on lifelong strict avoidance of fructose, sucrose, and sorbitol, which, when implemented early, leads to rapid clinical improvement and prevents irreversible organ damage. However, delayed diagnosis may result in unnecessary investigations, inappropriate treatments, and avoidable complications.

The aim of this report is to describe a late‐diagnosed case of HFI in a 12‐year‐old boy, highlighting the diagnostic challenges, key clinical and biochemical clues, and the importance of recognizing atypical presentations. The patient had a history of hypotonia, laryngomalacia, and persistent hepatomegaly who developed syncope during a fructose tolerance test after years of nonspecific gastrointestinal and hypoglycemic symptoms triggered by sweet food ingestion. Genetic analysis identified a homozygous pathogenic *ALDOB* variant, confirming the diagnosis. This case underscores the role of HFI as a true clinical “mimicker,” capable of presenting as chronic liver disease, malnutrition, or endocrine dysfunction. Early recognition of metabolic phenotype remains critical to ensure prompt dietary intervention and prevent long‐term complications.

## 2. Case Report

A 12‐year‐old boy was referred for evaluation of persistent hepatomegaly and recurrent gastrointestinal and hypoglycemic symptoms. He was born small for gestational age without postnatal catch‐up growth. His early medical history was significant for hypotonia and laryngomalacia, requiring follow‐up by pediatric otolaryngology. There was no known family history of metabolic, hepatic, or endocrine disorders, and no reported consanguinity.

During infancy, within the first year of life, he developed a severe episode of acute liver dysfunction characterized by jaundice, dark urine, and progressive hepatomegaly, requiring admission to a high‐dependency unit due to acute liver failure. Laboratory evaluation at that time revealed marked elevation of aminotransferases and cholestatic parameters. Serological testing was positive for IgM anti‐hepatitis A, while other infectious causes were excluded. Due to persistent hepatic abnormalities, a liver biopsy was performed, revealing a pattern of acute cholestatic hepatitis with Grade 2 mixed macro‐ and microvesicular steatosis. Although initially attributed to acute viral hepatitis, these histopathological findings are, in retrospect, suggestive of a possible underlying metabolic etiology.

From early childhood, his mother had observed recurrent episodes of postprandial vomiting, pallor, diaphoresis, and malaise following the ingestion of sweet foods, including fruits and processed products. These episodes were self‐limited but recurrent and were initially linked to a specific diagnosis. Throughout his life, he exhibited failure to thrive and intermittent hepatomegaly, leading to multidisciplinary evaluation by pediatric gastroenterology, hepatology, and endocrinology services, without identification of a unifying etiology.

**FIGURE 1:**
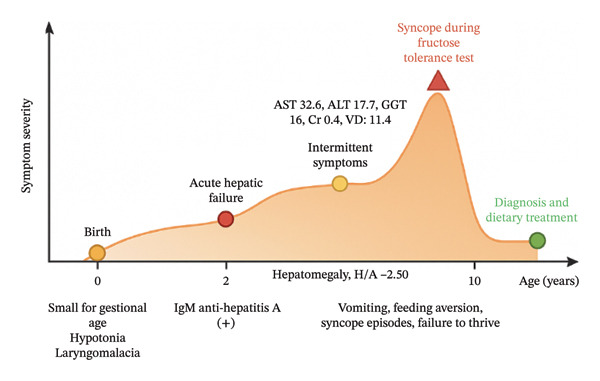
Clinical timeline: summary of the major clinical events and biochemical alterations across the disease course. Created by the authors.

At the age of 10 years, he underwent further evaluation. Physical examination revealed short stature (height‐for‐age Z score: −2.5 SD) and mild hepatomegaly, with no splenomegaly or stigmata of chronic liver disease. Laboratory studies showed aminotransferase levels at the upper normal limit, normal renal function, and vitamin D deficiency. Abdominal ultrasound revealed mild hepatic steatosis with diffuse parenchymal echogenicity, without biliary dilatation or splenomegaly (see Figure 1).

Given the history of reproducible symptoms after ingestion of sweet foods, a supervised fructose tolerance test was performed. Shortly after fructose administration, the patient developed diaphoresis, pallor, and loss of consciousness, corresponding to a severe hypoglycemic episode, raising strong suspicion of an inborn error of carbohydrate metabolism. Subsequently, targeted molecular analysis was performed through full sequencing of the *ALDOB* gene, which identified a homozygous pathogenic variant c.448G > C (p. Ala150Pro), classified as pathogenic according to the Human Genome Variation Society nomenclature, confirming the diagnosis of HFI.

Parental testing had not been performed at the time of reporting; however, carrier testing and genetic counseling were strongly recommended.

Following diagnosis, a strict lifelong diet free of fructose, sucrose, and sorbitol was instituted under multidisciplinary supervision. Subsequent follow‐up demonstrated marked clinical and biochemical improvement, with resolution of gastrointestinal and hypoglycemic symptoms, recovery of appetite and growth velocity, and normalization of hepatic enzymes and metabolic parameters. No further hypoglycemic episodes have been documented to date.

## 3. Discussion

HFI is a rare but potentially severe and life‐threatening metabolic disorder if not promptly recognized. It follows an autosomal recessive pattern, with an estimated global prevalence ranging between 1 in 10,000 and 1 in 20,000 live births. However, this figure is likely an underestimation, as milder or attenuated phenotypes frequently remain undiagnosed [4, 5]. In Latin America, reported cases remain sporadic, and diagnosis is often delayed or missed due to clinical overlap with functional gastrointestinal diseases or malnutrition. This diagnostic gap underscores the epidemiological importance of regional case documentation and molecular documentation, as each new report contributes to defining the mutation spectrum, improving clinical awareness and supporting the inclusion of HFI in future genetic screenings across underrepresented populations [4, 6].

Beyond its low prevalence, HFI reflects a complex interaction between genetics and metabolism, responsible for its wide clinical heterogeneity. Traditionally regarded as a paradigmatic example of how a single enzymatic defect can precipitate a multisystemic and reversible condition, contemporary understanding of HFI has expanded from a purely biochemical framework to a multilayered gene–phenotype model. In this paradigm, clinical heterogeneity is influenced not only by the nature of *ALDOB* mutations but also by population‐specific, environmental, and epigenetic factors that modulate residual enzyme activity and metabolic adaptation.

In recent years, advances in genetic sequencing technologies have broadened knowledge of the mutational spectrum of the *ALDOB* gene, revealing a wide range of pathogenic variants, including missense mutations, deletions, and splice‐site alterations, all contributing to the phenotypic variability observed. A recent systematic review by Pinheiros et al. reported 68 pathogenic variants identified across 1426 alleles, encompassing 85 distinct genotypes, and revealed marked ethnic and geographic clustering [4]. In Asian populations, a prevalence study demonstrated that approximately 82% of disease‐causing alleles correspond to globally recognized pathogenic variants, whereas 18% were unique or population‐specific [7]. In contrast, within European cohorts, the A150P (A149P), A174D, and N335K variants account for over 80% of pathogenic alleles, consistent with founder mutations exhibiting regional segregation [1, 8]. In the Latin American context, available data remain limited; however, a recent Brazilian cohort identified both classic pathogenic variants and previously undescribed mutations, highlighting the genetic diversity of regional populations and reinforcing the need to strengthen molecular registries to improve diagnostic sensitivity in underrepresented regions [6].

In our patient, targeted sequencing revealed a homozygous pathogenic variant *c.448G > C* (*p. Ala150Pro*) in *ALDOB,* located in Exon 5 and classified as pathogenic. This amino acid substitution alters a region of the enzyme Fructose‐1,6‐bisphosphate aldolase B, disrupting its secondary structure and significantly reducing its catalytic efficiency [9]. The *p.Ala150Pro* variant represents one of the most recurrent pathogenic alleles described in the HFI and particularly prevalent in the European and Latin American cohorts, supporting the hypothesis of a regional founder effect [4, 10].

From a genotype–phenotype perspective, missense variants such as *p. Ala150Pro* often retain residual enzymatic activity, which may explain milder or delayed presentations, as in the case of our patient, compared to nonsense or frameshift variants [11, 12]. Nevertheless, homozygous patients frequently present with the classic form of HFI, as described in greater detail later in this report [7].

This identification of recurrent *ALDOB* genes, such as *p. Ala150Pro,* highlights how molecular diagnosis can redefine prevention and management strategies in HFI. Although it is not currently included in standard newborn screening programs (mainly because no robust biochemical marker exists prior to fructose exposure), the growing implementation of genomic sequencing supports the feasibility of targeted genetic sequencing in families with known carriers or in populations where founder variations are common [13]. Early detection can avert inadvertent fructose exposure during infancy, leading to the prevention of severe hypoglycemia, hepatic dysfunction, and avoidable hospitalizations. Beyond its diagnostic utility, this genomic approach reinforces a translational shift in HFI management, from reactive treatment to proactive prevention, where cascade family testing, tailored dietary counseling, and integration of genetic information into national nutrition and pharmacovigilance, along with cascade family testing and structured patient education, represent a cost‐effective strategy, which could significantly improve safety, quality of life, and long‐term outcomes for affected individuals [14].

From a pathophysiological standpoint, recent advances have refined our understanding of the cellular events underlying HFI‐related hepatic injury. The aberrant activation of Adenosine monophosphate deaminase 2 (AMPD2) has emerged as a novel mechanism amplifying hepatic energy stress. Rather than direct metabolite accumulation alone, F1P appears to secondarily stimulate the AMPD2 pathway, leading to profound ATP depletion, nucleotide imbalance, mitochondrial dysfunction, and oxidative stress [15]. This energetic collapse translates clinically into hypoglycemia, hepatomegaly, and growth failure, hallmarks of HFI metabolic crisis. Collectively, these findings redefine HFI as a dynamic disorder of hepatic energy homeostasis, expanding potential avenues for targeted therapies aimed at metabolic modulation.

Along these lines, pharmacological inhibition of ketohexokinase (fructokinase) has emerged as a potential preclinical strategy to limit F1P formation, thereby reducing substrate load and attenuating metabolic injury. Such findings provide a more nuanced view of HFI, in which metabolic pathway modulation could complement traditional dietary management [16].

HFI displays remarkable phenotypic variability, largely determined by the age at exposure, amount of fructose intake, and the extent of residual Aldolase B activity. The disorder can range from severe, life‐threatening presentations in infancy to mild or late‐onset forms recognized in adolescence or adulthood [1, 17]. The classic infantile presentation typically arises with the introduction of fructose‐, sucrose‐, or sorbitol‐containing foods—often between four and 6 months of age—manifesting as postprandial vomiting, irritability, diaphoresis, lethargy, and hypoglycemia, which may progress to seizures or altered consciousness if unrecognized [18].

**FIGURE 2:**
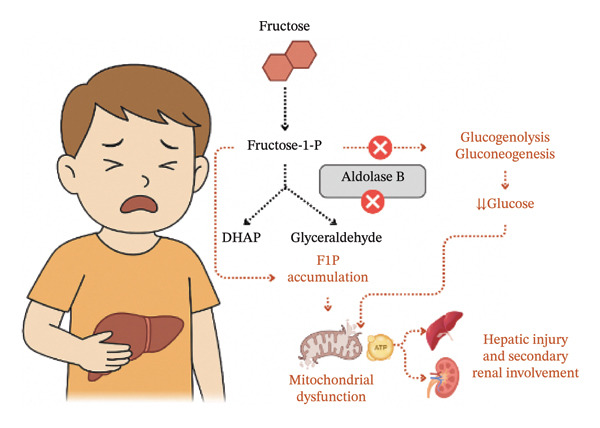
Pathophysiological mechanism of hereditary fructose intolerance: inhibition of Aldolase B and resulting systemic effects. Original figure created by the authors, adapted from [19].

The underlying hypoglycemia results from intracellular accumulation of F1P, which inhibits both gluconeogenesis and glycogenolysis, depleting ATP and disrupting hepatocellular energy metabolism. Consequently, hepatomegaly, mild transaminase elevation, and transient jaundice are common findings, which can evolve into hepatic failure if fructose exposure persists. Renal involvement, typically secondary to metabolic stress, reflects proximal tubular dysfunction, presenting as aminoaciduria and hypophosphatemia (see Figure 2) [19, 20].

In contrast, late‐onset or attenuated forms of HFI are explained by the presence of residual enzymatic activity of Aldolase B, which allows partial metabolism of fructose and prevents acute metabolic decompensation. In such cases, symptoms tend to be intermittent and subtle, typically consisting of postprandial abdominal pain, vomiting, and malaise, often accompanied by food avoidance behaviors, particularly toward fruits, juices, and desserts [3, 21].

The diagnostic approach relies on the integration of clinical, biochemical, and genetic data and requires a high index of clinical suspicion, especially in patients with mild, prolonged or nonspecific symptoms. In the present case, diagnosis was guided by a directed fructose tolerance test, which reproduced the patient’s characteristic symptoms and precipitated a syncopal episode due to hypoglycemia. This finding was pivotal in redirecting the diagnostic approach and subsequently justifying molecular confirmation. Although this presentation is unusual, it illustrates the importance of considering HFI even in atypical or late‐onset contexts, where clinical manifestations may be subtle and easily misattributed to functional gastrointestinal disorders or nonspecific food intolerances.

In retrospect, the episode initially attributed to acute hepatitis A during infancy may have represented an early manifestation of HFI, particularly given the temporal relationship with dietary exposures and the pattern of hepatic involvement. This raises the possibility that the condition was present from early life but remained unrecognized, leading to a delayed diagnosis. It is also important to consider that exposure to sorbitol‐containing medications or syrups may have acted as a trigger, as sorbitol is metabolized to fructose. Therefore, previously unexplained episodes of acute hepatic dysfunction in infancy could, in fact, represent metabolic decompensation in patients with HFI. Although symptoms were present early, the diagnosis was established later in childhood, and thus this case is best classified as a late‐diagnosed presentation rather than a true late‐onset form. Furthermore, certain clinical and laboratory features, such as recurrent vomiting following fructose ingestion, hypoglycemia, elevated transaminases, and the absence of a clear infectious etiology, should prompt consideration of HFI. With the widespread availability of molecular testing, invasive procedures such as liver biopsy have become largely unnecessary.

Definitive confirmation was achieved through molecular analysis of the *ALDOB* gene, which is currently regarded as the diagnostic gold standard for HFI. This technique identifies biallelic pathogenic variants responsible for Aldolase B deficiency, thereby eliminating the need for invasive procedures such as liver biopsy previously used for diagnosis. Beyond its diagnostic value, genetic testing enables accurate family counseling and facilitates early identification of carrier or asymptomatic relatives [22]. Parental segregation analysis has been initiated to clarify the inheritance pattern of the identified variant; however, results are currently pending. Taking into consideration the recessive nature of HFI, confirmation of parental carrier status is essential for accurate genetic interpretation and adequate counseling regarding future pregnancies. Notably, a truly de novo occurrence affecting both alleles would be exceedingly rare, further underscoring the importance of segregation analysis.

**FIGURE 3:**
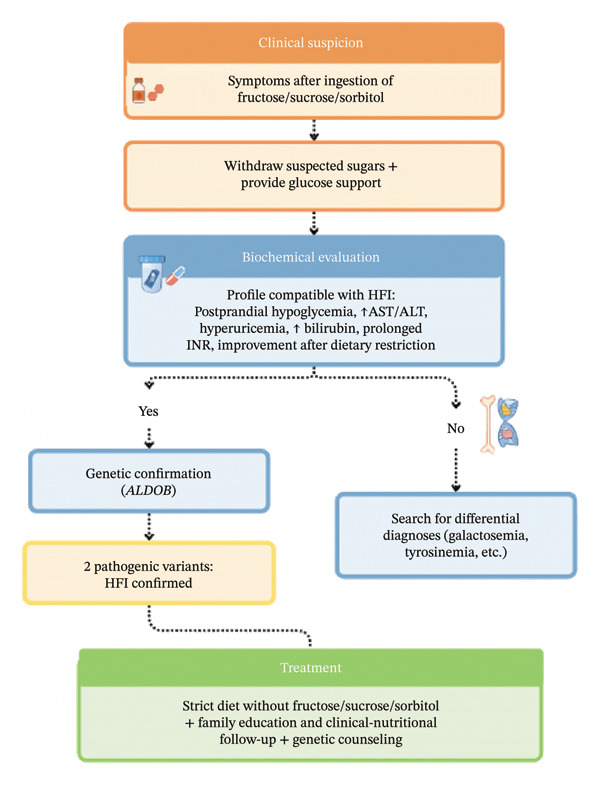
Proposed diagnostic algorithm for hereditary fructose intolerance: from clinical suspicion to molecular confirmation. Created by the authors, based on [1].

Figure 3 presents a diagnostic algorithm summarizing the key clinical, biochemical, and genetic features that support the suspicion, confirmation, and differentiation of HFI from other metabolic or gastrointestinal disorders.

The cornerstone of management in HFI continues to be the lifelong and absolute exclusion of fructose, sucrose, and sorbitol from the diet. This must be accompanied by meticulous nutritional supervision to prevent inadvertent ingestion from hidden sources, such as pharmaceutical excipients, processed foods, or sweetened supplements [22, 23]. When the disorder is recognized early and dietary compliance is rigorous, the clinical and biochemical prognosis is excellent, often allowing complete hepatic recovery, catch‐up growth, and normalization of metabolic parameters.

However, delayed diagnosis or unrecognized chronic exposure to fructose can lead to recurrent metabolic decompensation, hepatic fibrosis, and even progressive liver failure. These scenarios highlight the critical need for early molecular identification, lifelong patient education, and structured multidisciplinary follow‐up, integrating pediatric hepatology, gastroenterology, nutrition, and genetics to ensure sustained metabolic stability and optimal quality of life [21].

Despite the inherent limitations of a single‐patient report, this case contributes meaningfully to the literature by illustrating the atypical and prolonged diagnostic pathway of HFI, highlighting red flags for earlier recognition coupled, and underscoring the importance of avoiding hazardous provocative testing, to prevent dangerous fructose exposure in a vulnerable patient population.

## 4. Conclusion

HFI represents a diagnostic challenge in pediatrics due to its clinical heterogeneity and its ability to mimic a wide range of hepatic and metabolic disorders. The present case illustrates how a rare metabolic disease can remain unrecognized for years, initially manifesting as acute liver failure in infancy and later simulating other conditions, until a critical event—such as syncope secondary to fructose‐induced severe hypoglycemia—ultimately reveals its underlying cause.

Early recognition of warning signs such as persistent hepatomegaly, failure to thrive, and selective intolerance to sweet foods is crucial to raising suspicion before serious complications develop.

Genetic confirmation not only establishes a definitive diagnosis but also directs a highly effective and prognosis‐modifying treatment strategy.

“When fruit turns harmful” serves as a reminder that clinicians should consider HFI in patients with unexplained hepatic and gastrointestinal manifestations, even beyond infancy. Increased awareness and early diagnosis are critical to prevent misdiagnosis, avoid metabolic decompensation, and ensure excellent long‐term outcomes.

**Nomenclature**
HFI: Hereditary fructose intolerance
ALDOB: Aldolase B gene
HGVS: Human Genome Variation Society
ALT: Alanine aminotransferase
AST: Aspartate aminotransferase
SD: Standard deviation

## Author Contributions

Michelle Higuera Carrillo and Sara Vallejo Tamayo contributed equally to the conception, clinical follow‐up, and drafting of the manuscript. Michelle Higuera Carrillo performed the literature review and coordinated the diagnostic and genetic confirmation process. Sara Vallejo Tamayo contributed to the clinical data collection, translation of medical terminology, and final manuscript editing. Dr. Melquisedec Vargas Sandoval supervised the patient’s clinical management, provided expert guidance during the diagnostic workup, and critically reviewed the final version of the manuscript for clinical accuracy.

## Funding

This research did not receive any specific grant from funding agencies in the public, commercial, or not‐for‐profit sectors.

## Disclosure

All authors read and approved the final manuscript.

## Ethics Statement

Written informed consent for participation, publication, and for the use of anonymized clinical information was obtained from the patient’s legal guardian.

## Consent

Please see the Ethics Statement.

## Conflicts of Interest

The authors declare no conflicts of interest.

## Data Availability

The data that support the findings of this study are available on request from the corresponding author. The data are not publicly available due to privacy or ethical restrictions.

## References

[1] Singh S. K. and Sarma M. S. , Hereditary Fructose Intolerance: A Comprehensive Review, WJCP. (2022) 11, no. 4, 321–329, 10.5409/wjcp.v11.i4.321. 36052111 PMC9331401

[2] Bouteldja N. and Timson D. J. , The Biochemical Basis of Hereditary Fructose Intolerance, Journal of Inherited Metabolic Disease. (2010) 33, no. 2, 105–112, 10.1007/s10545-010-9053-2, 2-s2.0-77953231022. 20162364

[3] Kim A. Y. , Hughes J. J. , Pipitone Dempsey A. , Sondergaard Schatz K. , Wang T. , and Gunay-Aygun M. , Pitfalls in the Diagnosis of Hereditary Fructose Intolerance, Pediatrics. (2020) 146, no. 2, 10.1542/peds.2019-3324. 32709737

[4] Pinheiro F. C. , Sperb‐Ludwig F. , and Schwartz I. V. D. , Epidemiological Aspects of Hereditary Fructose Intolerance: A Database Study, Human Mutation. (2021) 42, no. 12, 1548–1566, 10.1002/humu.24282. 34524712

[5] Buziau A. M. , Schalkwijk C. G. , Stehouwer C. D. A. , Tolan D. R. , and Brouwers M. C. G. J. , Recent Advances in the Pathogenesis of Hereditary Fructose Intolerance: Implications for Its Treatment and the Understanding of Fructose-Induced Non-Alcoholic Fatty Liver Disease, Cellular and Molecular Life Sciences. (2020) 77, no. 9, 1709–1719, 10.1007/s00018-019-03348-2. 31713637 PMC11105038

[6] Tang M. , Chen X. , Ni Q. et al., Estimation of Hereditary Fructose Intolerance Prevalence in the Chinese Population, Orphanet Journal of Rare Diseases. (2022) 17, no. 1, 10.1186/s13023-022-02487-3. PMC9419342 36028839

[7] Davit-Spraul A. , Costa C. , Zater M. et al., Hereditary Fructose Intolerance: Frequency and Spectrum Mutations of the Aldolase B Gene in a Large Patients Cohort From France—Identification of Eight New Mutation, Molecular Genetics and Metabolism. (2008) 94, no. 4, 443–447, 10.1016/j.ymgme.2008.05.003, 2-s2.0-46949110730. 18541450

[8] Coffee E. M. , Yerkes L. , Ewen E. P. , Zee T. , and Tolan D. R. , Increased Prevalence of Mutant Null Alleles That Cause Hereditary Fructose Intolerance in the American Population, Journal of Inherited Metabolic Disease. (2010) 33, no. 1, 33–42, 10.1007/s10545-009-9008-7, 2-s2.0-77649234518. 20033295 PMC2954661

[9] Cross N. C. P. , Cox T. M. , De Franchis R. et al., Molecular Analysis of Aldolase B Genes in Hereditary Fructose Intolerance, Lancet. (1990) 335, no. 8685, 306–309, 10.1016/0140-6736(90)90603-3, 2-s2.0-0025060128. 1967768

[10] Santer R. , Rischewski J. , Von Weihe M. et al., The Spectrum of Aldolase B (ALDOB) Mutations and the Prevalence of Hereditary Fructose Intolerance in Central Europe, Human Mutation. (2005) 25, no. 6, 10.1002/humu.9343, 2-s2.0-34548094022. 15880727

[11] Kim M. S. , Moon J. S. , Kim M. J. , Seong M. W. , Park S. S. , and Ko J. S. , Hereditary Fructose Intolerance Diagnosed in Adulthood, Gut and Liver. (2021) 15, no. 1, 142–145, 10.5009/gnl20189. 33028743 PMC7817925

[12] Ferri L. , Caciotti A. , Cavicchi C. et al., Integration of PCR-Sequencing Analysis With Multiplex Ligation-Dependent Probe Amplification for Diagnosis of Hereditary Fructose Intolerance. En: SSIEM, JIMD Reports-Case and Research Reports, 2012/3, 2012, Springer Berlin Heidelberg, 31–37, 10.1007/8904_2012_125, 2-s2.0-84902721634. PMC3565637 23430936

[13] James C. L. , Rellos P. , Ali M. , Heeley A. F. , and Cox T. M. , Neonatal Screening for Hereditary Fructose Intolerance: Frequency of the Most Common Mutant Aldolase B Allele (A149P) in the British Population, Journal of Medical Genetics. (1996) 33, no. 10, 837–841, 10.1136/jmg.33.10.837. 8933337 PMC1050763

[14] Beigh M. , Next-Generation Sequencing: The Translational Medicine Approach From Bench to Bedside to Population, Medicines. (2016) 3, no. 2, 10.3390/medicines3020014. PMC5456221 28930123

[15] Andres-Hernando A. , Orlicky D. J. , Kuwabara M. et al., Activation of AMPD2 Drives Metabolic Dysregulation and Liver Disease in Mice With Hereditary Fructose Intolerance, Communications Biology. (2024) 7, no. 1, 10.1038/s42003-024-06539-1. PMC11239681 38992061

[16] Koene E. J. C. , Buziau A. M. , Cassiman D. et al., Safety and Efficacy of Pharmacological Inhibition of Ketohexokinase in Hereditary Fructose Intolerance, Journal of Clinical Investigation. (2025) 135, no. 6, 10.1172/JCI187376. PMC11910217 39932807

[17] Arica V. , Yildiz E. G. Ö. , Kök S. et al., Current Therapeutic Approaches in Infantile Colic: A Comprehensive Review, Turkish Archives of Pediatrics. (2025) 10.5152/TurkArchPediatr.2025.25388. 42228814

[18] Ali M. , Rellos P. , and Cox T. M. , Hereditary Fructose Intolerance, Journal of Medical Genetics. (1998) 35, no. 5, 353–365, 10.1136/jmg.35.5.353. 9610797 PMC1051308

[19] Hwang J. J. , Jiang L. , Hamza M. et al., The Human Brain Produces Fructose from Glucose, JCI Insight. (2017) 2, no. 4, https://insight.jci.org/articles/view/90508, 10.1172/jci.insight.90508. PMC5313070 28239653

[20] Febbraio M. A. and Karin M. , Sweet Death: Fructose as a Metabolic Toxin that Targets the Gut-Liver Axis, Cell Metabolism. (2021) 33, no. 12, 2316–2328, 10.1016/j.cmet.2021.09.004. 34619076 PMC8665123

[21] Kim M. S. , Moon J. S. , Kim M. J. , Seong M. W. , Park S. S. , and Ko J. S. , Hereditary Fructose Intolerance Diagnosed in Adulthood, Gut and Liver. (2021) 15, no. 1, 142–145, 10.5009/gnl20189. 33028743 PMC7817925

[22] Úbeda F. , Santander S. , and Luesma M. J. , Clinical Practice Guidelines for the Diagnosis and Management of Hereditary Fructose Intolerance, Diseases. (2024) 12, no. 3, 10.3390/diseases12030044. PMC10969590 38534968

[23] Li H. , Byers H. M. , Diaz-Kuan A. et al., Acute Liver Failure in Neonates With Undiagnosed Hereditary Fructose Intolerance due to Exposure From Widely Available Infant Formulas, Molecular Genetics and Metabolism. (2018) 123, no. 4, 428–432, 10.1016/j.ymgme.2018.02.016, 2-s2.0-85042732053. 29510902

